# Pulmonary endothelial cells from different vascular segments exhibit unique recovery from acidification and Na^+^/H^+^ exchanger isoform expression

**DOI:** 10.1371/journal.pone.0266890

**Published:** 2022-05-03

**Authors:** Dylan Adams, Chung-Sik Choi, Sarah L. Sayner

**Affiliations:** 1 Department of Physiology and Cell Biology, University South Alabama, College of Medicine, Mobile, Alabama, United States of America; 2 Center for Lung Biology, University of South Alabama, College of Medicine, Mobile, Alabama, United States of America; Ann and Robert H Lurie Children’s Hospital of Chicago, Northwestern University, UNITED STATES

## Abstract

Sodium-hydrogen exchangers (NHEs) tightly regulate intracellular pH (pH_i_), proliferation, migration and cell volume. Heterogeneity exists between pulmonary endothelial cells derived from different vascular segments, yet the activity and isoform expression of NHEs between these vascular segments has not been fully examined. Utilizing the ammonium-prepulse and recovery from acidification technique in a buffer lacking bicarbonate, pulmonary microvascular and pulmonary artery endothelial cells exhibited unique recovery rates from the acid load dependent upon the concentration of the sodium transport inhibitor, amiloride; further, pulmonary artery endothelial cells required a higher dose of amiloride to inhibit sodium-dependent acid recovery compared to pulmonary microvascular endothelial cells, suggesting a unique complement of NHEs between the different endothelial cell types. While NHE1 has been described in pulmonary endothelial cells, all NHE isoforms have not been accounted for. To address NHE expression in endothelial cells, qPCR was performed. Using a two-gene normalization approach, *Sdha* and *Ywhag* were identified for qPCR normalization and analysis of NHE isoforms between pulmonary microvascular and pulmonary artery endothelial cells. NHE1 and NHE8 mRNA were equally expressed between the two cell types, but NHE5 expression was significantly higher in pulmonary microvascular versus pulmonary artery endothelial cells, which was confirmed at the protein level. Thus, pulmonary microvascular and pulmonary artery endothelial cells exhibit unique NHE isoform expression and have a unique response to acid load revealed through recovery from cellular acidification.

## Introduction

Pulmonary endothelial cells separate the blood from the underlying tissue. In addition to acting as a semipermeable barrier, pulmonary endothelial cells have a multitude of other functions including regulation of coagulation, inflammation and angiogenesis. At a cellular level, many of these processes have been linked to the transport activity of sodium-hydrogen exchangers (NHEs), which tightly regulate intracellular pH (pH_i_) [1, 2]. Importantly, small changes in pH_i_ affect the activity of weak acids and bases, such as peptides and proteins, that depend on a narrow pH range in order to maintain their structure and function; thus, most cells robustly regulate pH_i_ [3, 4]. Currently, the mechanisms whereby pulmonary endothelial cells maintain pH_i_ is not fully resolved.

Functional heterogeneity exists between pulmonary endothelial cells from different vascular segments, such that pulmonary microvascular endothelial cells (PMVECs), sourced from pulmonary capillaries, have unique cellular features compared to pulmonary artery endothelial cells (PAECs), sourced from pulmonary arteries, and pulmonary vein endothelial cells [5–12]. For example, PMVECs form a more restrictive barrier to solutes compared to PAECs [13, 14]. PMVECs are highly proliferative and utilize aerobic glycolysis leading to acidification of the media, while PAECs, which are not as proliferative, utilize aerobic respiration and do not acidify the media. In addition, PMVECs have proangiogenic phenotype compared to PAECs [11, 12, 15]. At the signaling level, the response to calcium agonists, and cAMP generation are unique between endothelial cells from these different vascular segments [16–20]. Furthermore, there is emerging evidence that PMVECs and PAECs exhibit unique pH_i_ regulation [21].

Cytosolic pH tends to be acidic due to the negative electrical potential inside the cell and acid equivalence generated through various metabolic processes [4, 22, 23]. In order to stringently regulate pH_i_, protons are extruded (acid extruders) from the cell by exchangers (antiporters) and cotransporters (symporters). Alkali-cation-H^+^ exchangers directly couple the transfer of H^+^ across a biological membrane to the counter current of Na^+^ or K^+^. Thirteen Na^+^/H^+^ exchangers are classified as SLC9A1-9 (NHE1-9), SLC9B1-2 (NHA1-2) and SLC9C1-2, and are expressed at the plasma membrane and/or on organelles along the secretory and endocytic pathways [24]. In addition, both lactate-H^+^ cotransporters and Na^+^-bicarbonate cotransporters (NBCs) act as acid extruders: lactate-H^+^ cotransporters mediate the extrusions of H^+^ driven by the outward lactate gradient, while NBCs mediate the uptake of extracellular bicarbonate coupled to the influx of Na^+^ down its concentration gradient [24, 25]. Beside acid extruders, cells also express acid loaders such as anion exchangers (AEs) in which the inward Cl^-^ gradient drives HCO_3_^-^ efflux to alkalinize the cell [25]. Tightly coupled to the movement of HCO_3_^-^ across the membrane is its generation by carbonic anhydrase (CA), which catalyzes the interconversion of CO_2_ and HCO_3_^-^ [26]. Isoform expression of NBCs and CA has been described in PMVECs and PAECs [21, 27]. NHE1 has been implicated in regulation of pH_i_ in pulmonary endothelium, yet a full evaluation of the growing family of NHEs expressed at the plasma membrane has not been completed [28–31]. Thus, here we sought to identify NHEs in PMVECs and PAECs.

At the cellular level, acid extruder transporter activity can be resolved by monitoring the recovery from intracellular acidification, which is established using the ammonium prepulse technique [4, 32]. To enable detection of NHEs, a buffering system excluding bicarbonate at all steps of the recovery phase is employed. NHEs have isoform sensitivity to amiloride; therefore, NHE isoforms can be resolved using a sodium-amiloride buffering system. Following the ammonium prepulse, PMVECs and PAECs exhibited a unique amiloride-sensitive response to recovery from acidification. Quantitative PCR supported by western analysis further revealed a distinct NHE isoform expression profile between PMVECs and PAECs. Thus, these studies corroborate heterogeneity between pulmonary endothelial cells from different vascular segments highlighting the unique manner in which PMVECs and PAECs handle an acid load.

## Materials and methods

### Isolation of pulmonary endothelial cells

Pulmonary endothelial cells were obtained from the Cell Culture Core, Center for Lung Biology at the University of South Alabama. Isolation, characterization, culturing and routine passaging has been described in detail previously [14, 16, 33–35].

In brief, male Sprague-Dawley rats (CD strain, 350–400 g; Charles River) were anesthetized by an intraperitoneal injection of pentobarbital sodium (50 mg/Kg, Nembutal, Abbott Laboratories, Chicago, IL). A sternotomy was performed, and the heart and lungs excised *en bloc* and placed into a bath containing ice-cold DMEM (catalogue no. 11965, Gibco). For isolation of PAECs, the pulmonary artery was dissected from the hilus down to second generation vessels, inverted and endothelial cells obtained by gentle intimal scraping with a plastic cell lifter. Harvested primary cells were strained with a 20 μM filter (BD Biosciences) seeded into a 100-mm petri dish containing seeding medium (∼1:1 DMEM-Ham’s F-12 [catalogue no. 11765054, Gibco], 20% heat inactivated FBS [catalogue no S11550H, Atlanta Biologicals] and 100 U/mL penicillin and 100 μg/mL streptomycin [catalogue no. 15070063, Gibco]). For isolation of PMVECs, once the lungs were cleared of blood, flow was established with a physiological salt solution containing 50 mg of hyaluronidase I and collagen IV-coated 50-μm microspheres. Perfusate flow alternated between anterograde and retrograde (0.03 ml ⋅ min−1 ⋅ g−1), and the effluent endothelial cell bound microbeads collected on ice. The microbeads with primary PMVECs were washed three times with RPMI 1640 medium (catalogue no. 21875034, Gibco) containing 25% FBS and resuspended and cultured in RPMI media containing 20% rat serum, 0.1% gentamicin, and EC-conditioned medium (2:1). All cells were cultured in a humidified chamber (Forma 3130 CO_2_ HEPA Incubator, Thermal Scientific) at 37°C adjusted to 5% CO_2_. When PAEC and PMVEC primary cultures reached confluence, cells were passaged by trypsin digest into EC growth media (DMEM, 10% FBS and 1% Penicillin-streptomycin) and routinely cultured, as described previously [27, 36–39]. Separate “n” values represent independent seeding event from a unique parent dish.

At confluence, the endothelial cells demonstrated a cobblestone morphology. Endothelial phenotype was confirmed by uptake of 1,1′-dioctadecyl-3,3,3′,3′-tetramethylindocarbocyanine-labeled low-density lipoprotein (DiI-acetylated low-density lipoprotein), expression of classical endothelial markers (Factor VIII-Rag, CD31 [PECAM], eNOS and CD144 [VE-cadherin] and Von Willebrand factor [VWF]), and ability to form networks on Matrigel [11]. Discrimination between the two endothelial cell types was confirmed by lectin binding: while PMVECs bind *Griffonia simplicifolia* but not *Helix pomatia*, PAECs bind *Helix pomatia* but not *Griffonia simplicifolia*. Absence of smooth muscle cells is confirmed by the absence of immunostaining with smooth muscle α-actin antibodies.

All experimental procedures were performed in accordance with current provisions of the US Animal Welfare Act and were approved by the Institutional Animal Care and Use Committee of the University of South Alabama.

### Measurement of intracellular pH (pH_i_)

PAECs and PMVECs were seeded onto 35 mm MatTek culture dishes (part no P35G-1.5-20-C, MatTeK corporation). Upon confluence, culture media was removed, and cells washed twice in Hank’s Balanced Salt Solution (HBSS, catalogue no. 14025, Invitrogen). Cells were incubated in HBSS containing the membrane-permeable pH sensitive fluorescent indicator dye, 2’,7’-Bis-(2-Carboxyethyl)-5-(and-6)-Carboxyfluorescein, Acetoxymethyl Ester (0.1 μM, BCECF-AM, catalogue no. B1170, Invitrogen) for 30 minutes at 5% CO_2_ and 37°C. After incubation, cells were washed twice in HBSS to remove excess dye and incubated for an additional 15 minutes at 37°C in HBSS to allow complete de-esterification of cytosolic dye. Following de-esterification of the dye, acquisition of the data occurred at room temperature (20°C). BCECF-AM loaded PAECs and PMVECs were monitored with an Olympus IX70 inverted microscope at 40X with a xenon arc lamp photomultiplier system, and data acquired and analyzed with PTI Felix software (Photon Technology International Ltd.). Cells were excited at 490 nm (pH sensitive) and 440 nm (pH insensitive) and emissions collected at 535 nm. Measurements were performed at 7 s intervals.

### Ammonium prepulse

Following BCECF-AM loading, baseline pH_i_ was established in HBSS solution. Upon stable baseline, cells were exposed to the NH_4_Cl prepulse followed by removal of NH_4_Cl to induce rapid intracellular acidification using the CO_2_/HCO_3_^-^ free and Na^+^ free buffer. In the presence of amiloride (catalogue no. 129876, Sigma) and absence of Na^+^, negligible pH_i_ recovery is expected with this buffer. Once the Na^+^ free buffer is replaced with the CO_2_/HCO_3_^-^ free Na^+^-containing buffer and in the absence of amiloride, complete recovery from acidification occurs. Following complete recovery, a pH calibration curve was established for each experiment using the mean 490/440 nm ratio of KCl-nigericin buffering solutions, composed of 10 μM nigericin (catalogue no. N7143, Sigma), 105 mM KCl, 1 mM MgCl_2_, 1.5 mM glucose, and either 20 mM 4-(2-hydroxyethyl)-1-piperazineethanesulfonic acid (HEPES), or 20 mM Tris adjusted to a pH range of 6.5 to 7.5 or 8.0 to 8.5, respectively at room temperature. Experimental ratio measurements (490/440 nm) were compared to the nigericin calibration curve to generate pH_i_ values.

### Lysis, purification of total RNA and cDNA synthesis

At confluence, PMVEC or PAEC lysate, and whole kidney and stomach tissue was prepared using the RNeasy Mini Kit (Qiagen) according to the manufacturer’s instructions and lysates stored at -80°C. Following extraction, total RNA was treated with DNase I to remove genomic DNA contamination. The concentration and integrity of the RNA samples was assessed by measuring spectral absorption at 260 and 280nm using NanoDrop Lite spectrophotometer (Thermo Scientific). An A_260/A280_ estimate of 1.8–2.2 was accepted. cDNA was synthesized from 1 μg of DNA-free RNA template in a 20 μL reaction volume using iScript cDNA Synthesis Kit (catalogue no 1708891, Bio-Rad). The complete reaction mixture was incubated in a Mastercycler gradient (Eppendorf) at 25°C for 5 min followed by 20 min at 46°C for reverse transcription. The reverse transcriptase was inactivated by heating to 95°C for 1 min. Samples of cDNA were stored at -20°C.

### Real-time quantitative PCR (qPCR)

Real-time quantitative PCR (qPCR) reactions were performed in 96 well PCR plates (BioRad) using a BioRad CFX Connect thermal cycler and CFX Manager software (CFX Connect Real-Time PCR Detection System; BioRad). Each 20 μL qPCR reaction contained 2 x supermix (iQ™ SYBR® Green Supermix, catalogue no 170880, Bio-Rad), 300 nM forward and reverse primers, and 1μL of sample cDNA template. A master mix containing cDNA, 2x SYBR Green Supermix, and water was made for each sample to reduce pipetting errors. The thermal cycling conditions were as follows: 95°C for 180 s followed by 40 cycles at 95°C for 20 s, 60°C for 20 s, and 72°C for 30 s. In all cases, a final dissociation melting curve step of 15 s at 95°C, 60 s at 60°C, followed by a gradual increase in temperature to 95°C was performed to verify the specificity of each reaction. Two or three technical replicates were included for each biological sample. All biological samples to be tested for each primer pair were included on the same plate to obviate the need for inter-plate calibration. Nuclease-free water without sample template was included in duplicate as a negative control for each primer pair. CFX Connect Manager software calculated quantification cycle (Cq) data. The final PCR products was separated using 2% agarose gel electrophoresis to further verify primer specificity, rule out genomic DNA contamination, and significant primer-dimer contribution.

### Primer design and verification and selection

Previously verified primers for rat (*Rattus norvegicus*) succinate dehydrogenase complex (*Sdha*), tyrosine 3-monooxygenase (*Ywhag*), phosphoglycerate kinase 1 (*Pgk1*), peptidylprolyl isomerase A (*Ppia*), ribosomal protein L13A (*Rpl13A*), and sodium-hydrogen exchanger 8 (*Nhe8*) were obtained from the literature [40–42]. Primers for rat, hypoxanthine phosphoribosyltransferase 1 (*Hprt1*), glyceraldehyde-3-phosphate dehydrogenase (*Gapdh*), and β-actin (*Actb*, Table 1) and *Nhe1-5* were designed using Primer-Blast (http://www.ncbi.nlm.nih.gov/tools/primer-blast/) to specifically cross intron-exon junctions.

**Table 1:** Primers for candidate reference genes with exon boundaries and amplicon size.

Gene	Chromosome Exon boundary	Primer Sequences (5’→3’)	Amplicon Length (bp)
*Sdha*	Chromosome 1	F: CTCTTTTGGACCTTGTCGTCTTT	102
	Exons 10–11	R: TCTCCAGCATTTGCCTTAATCGG	
*Ywhag*	Chromosome 12	F: TTCCTAAAGCCCTTCAAGGCA	100
	Exons 1–2	R: GGCTTTCTGCACTAGTTGCTCG	
*Pgk1*	X Chromosome	F: GAAGGGAAGGGAAAAGATGC	180
	Exons 4–6	R: AAATCCACCAGCCTTCTGTG	
*Ppia*	Chromosome 14	F: CCAAACACAAATGGTTCCCAGT	135
	Exons 4–5	R: ATTCCTGGACCCAAAACGCT	
*Hprt1*	X Chromosome	F: AGTCCCAGCGTCGTGATTAGTGAT	139
	Exons 1–3	R: GAGCAAGTCTTTCAGTCCTGTCCA	
*Actb*	Chromosome 12	F: CGTTGACATCCGTAAAGAC	178
	Exon 5–6	R: ATAGAGCCACCAATCCAC	
*Gapdh*	Chromosome 4	F: GGTGAAGGTCGGTGTGAACGGATT	502
	Exon 2–5	R: GATGCCAAAGTTGTCATGGATGAC	
*Rpl13a*	Chromosome 1	F: GGATCCCTCCACCCTATGACA	130
	Exons 5–7	R: CTGGTACTTCCACCCGACCTC	

### Reference gene selection

Eight reference genes, commonly used in the literature, were selected to determine the optimal reference gene for comparison between PMVECs and PAECs. *Sdha* and *Ywhag* were specifically included, because stable expression in whole rat lung tissue has been described previously [43]. The genes were evaluated in duplicate for all cDNA samples of both PMVECs and PAECs (n = 5). Following qPCR, resulting Cq data was exported as a Microsoft Excel spreadsheet directly into qBase^PLUS^ (Biogazelle NV, Belgium), a qPCR analysis program with built in features for identification of stably expressed reference genes, normalization, post-PCR quality control, inter-run calibration, and statistical analysis of target gene expression. The geNorm algorithm, described by Vandesompele *et al*., is incorporated into qBase^PLUS^ software for identification of stably expressed reference genes [44]. The algorithm initially calculates the average variation of one reference gene compared with all other reference genes and is represented by the internal control expression stability measure, M. If two reference genes are stably expressed between different samples (i.e. PAECs and PMVECs), then M will not change regardless of these experimental conditions; thus, lower M values reflect more stable reference gene expression. Subsequently, the difference of the average Cq value (ΔCq) between PAECs and PMVECs for each reference gene was calculated and normalized to M (ΔCq*M) [45]. Finally, in order to determine the number of reference genes required in the analysis, the geometric means of two sequential normalization factors (NF_n_ + NF_n+1_) was calculated according to the geNorm algorithm. In this process, the two most stable reference genes, according to M values, were evaluated with pairwise variation of normalization factors against the top three most stable reference genes (V2/3). Then, the next most stable reference gene was added to the equation (V3/4) until all genes were evaluated. A threshold level of 0.15 was selected since the benefit of adding additional reference genes once the V-value drops below 0.15 is limited [44].

### Normalization of target gene expression

qBase^PLUS^ normalizes raw qPCR Cq data to reference gene expression by initially calculating the mean and standard deviation of all technical replicates, which are then converted to relative quantities based on gene specific amplification efficiencies. A sample specific normalization factor is determined by calculating the geometric mean of the reference genes. Relative quantities are normalized by dividing by this normalization factor. Normalized values were imported into GraphPad Prism for graphing.

### Statistical analysis using qBase^PLUS^ software

Normalized relative expression was compared between PMVECs and PAECs for each NHEs using an unpaired *t*-test. This analysis was performed within qBase^PLUS^ and exported to GraphPad Prism for graphing.

### Western blot analysis

Frozen rat brain tissue was pulverized over liquid nitrogen and the resulting homogeneous powder stored at -80°C. The pulverized tissue was lysed in ice cold lysis buffer (phosphate buffered RIPA [Boston BioProducts, catalogue #BP-415] with HALT phosphatase and protease inhibitor cocktail [Thermo Scientific, Rockfort, IL, Ref #1861280]), homogenized (3 times for 10 seconds each with 30 second interval, Tissumizer, Tekmar Co., Cincinnati, Oh), followed by sonication (2 times 10 seconds each with 10 seconds interval, VirSonic 60, Virtis Co, NY). The lysate was rotated for 30 minutes at 4°C and centrifuged at 14,000 rpm for 15 minutes at 4°C. The supernatant was collected and protein assay performed as described previously and following manufactures instructions [46]. PMVECs and PAECs were grown to confluence in 60 mm dishes, rinsed in ice-cold PBS and lysed in lysis buffer, as described previously [38]. Samples were adjusted to equal protein concentration, sample buffer (Thermoscientific, 4X LDS sample buffer catalogue # B0007) added and samples heated to 37°C for 30 minutes. Proteins were resolved by SDS-PAGE (Thermofisher Scientific, Bolt 4–12% Bis-Tris Plus gels, catalogue no. NP0322) and proteins transferred to nitrocellulose membranes. Membranes were incubated with blocking buffer (Tris-buffered saline [13 mM NaCl and 25 mM Tris, pH 7.4] supplemented with 0.5% Tween 20 (TTBS) and 5% nonfat dry milk). Membranes were incubated with NHE5 (Kind gift Dr. Orlowski, Vancouver, Canada) or tubulin (Sigma, catalogue no. T6199) antibody overnight at 4°C in TTBS containing 1% nonfat dry milk. Membranes were rinsed (3 times 10 minutes) in TTBS and incubated in IgG (H+L) conjugated to horseradish peroxidase secondary antibody (Jackson ImmunoResearch Inc, West Grove PA; goat anti-rabbit catalogue no. 111-035-144 and goat anti-mouse catalogue no. 111-035-146, respectively) for 1 hour at room temperature. Finally, membranes were rinsed in TTBS and chemiluminescence of proteins was visualized with SuperSignal^TM^ West Atto ultimate or SuperSignalTM West femto, respectively (Thermo Scientific; catalogue nos. A38554 and 34094) and detected with ChemiDoc Imaging System (BioRad). Densitometry was performed on western blots using ImageJ.

### Data analysis using prism GraphPad

GraphPad Prism software (GraphPad Prism version 9.00, GraphPad Software, La Jolla California USA, www.graphpad.com) was used for statistical analysis (unpaired *t*-test or two-way ANOVA with Bonferroni post-hoc test as appropriate) and graphing of ratiometric data attained from PTI Felix software. In addition, GraphPad Prism was used to prepare graphs from qBase^PLUS^ geNorm results.

## Results

### Recovery from intracellular acidification

**Fig 1:**
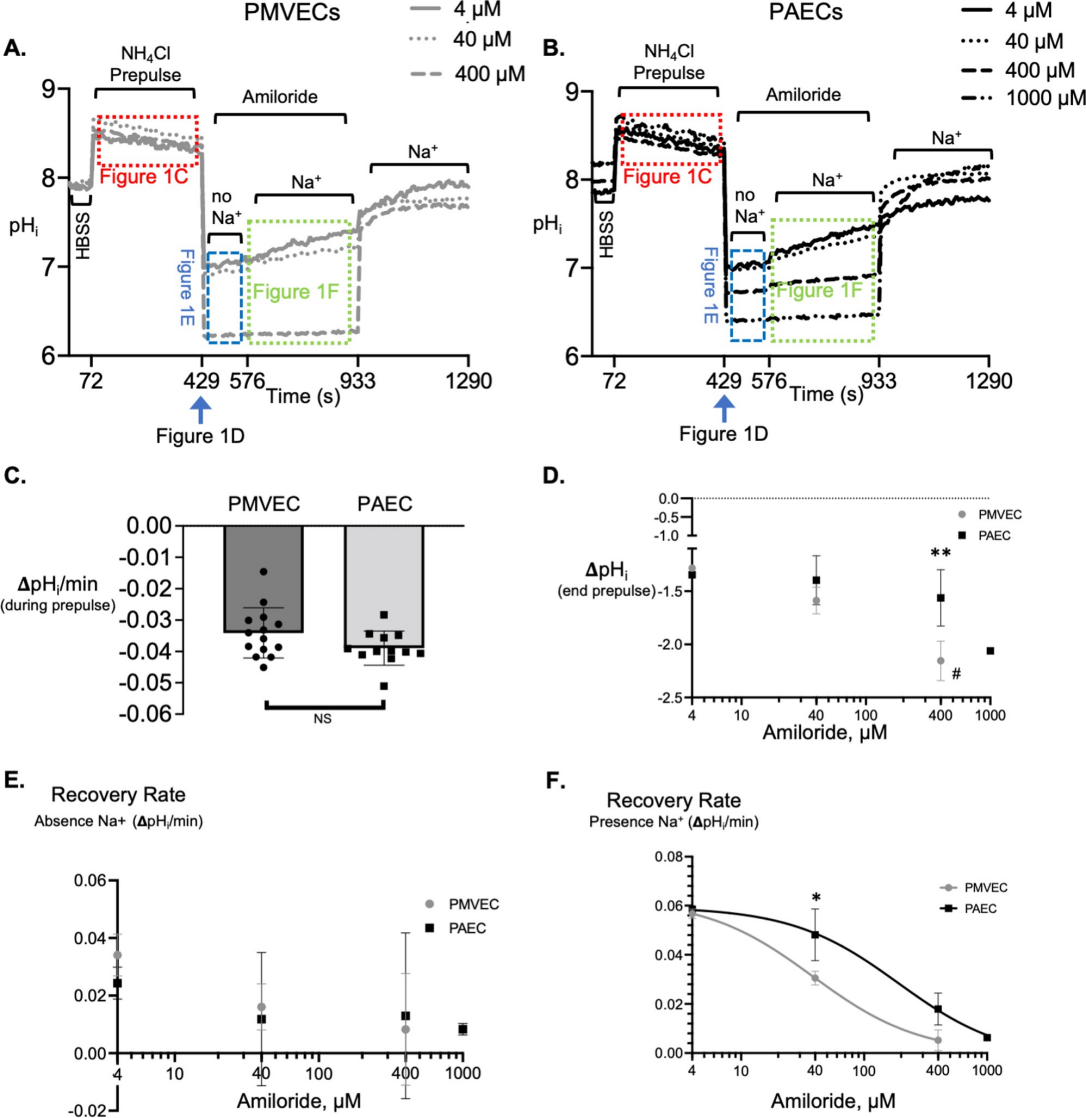
PMVECs and PAECs demonstrate different amiloride sensitivity during recovery from cellular acidification. Traces showing the pH_i_ following the ammonium prepulse and recovery from acidification in the presence of increasing doses of amiloride performed on BCECF-loaded PMVECs (A) and PAECs (B). Following HBSS baseline, the NH_4_Cl technique was used to induce cellular alkalinization. During the NH_4_Cl phase (red box in Panels A and B, summarized in C), the slow and gradual acidification was similar between PMVECs and PAECs. Upon removal of the NH_4_Cl prepulse, there was an amiloride-dose dependent decrease in pH_i_ (blue arrow Panels A and B, summarized in D), followed by a negligible recovery in pH_i_ in the presence of amiloride but absence of Na^+^ (blue box Panels A and B, summarized in E). Once Na^+^ was returned to the extracellular buffer, PMVECs and PAECs demonstrate a unique amiloride dose-dependent rate of recovery from acidification (green box Panels A and B, summarized in F). NS not significant; **P<0.001 PMVECs versus PAECs; #P<0.001 PMVECs 40 μM versus 400 μM amiloride; *P<0.05 PMVECs versus PAECs; n = 3–6 experiments for each amiloride dose in PMVECs and PAECs.

Cellular transporters necessary to regulate pH_i_ can be evaluated through the ammonium prepulse technique, in which ammonium salts are used to induce pH_i_ transients (Fig 1A and 1B). This protocol, commonly used to detect expression and functional activity of NHEs [4, 22, 32, 44, 47, 48], can be described in three phases.

The first phase begins by establishing a baseline pH_i_ in HBSS (Table 2). In the absence of CO_2_, pH_i_ is more alkaline than would be expected [4]; therefore, this is not a good surrogate for recording absolute pH_i_. Once a stable baseline is established, the external solution is exchanged for an ammonium chloride solution (NH_4_Cl prepulse, Table 2) in which an equilibrium exists ${NH}_{4}^{+}⇋{NH}_{3}+H^{+}$. As first demonstrated by Jacobs [49], once HBSS is exchanged for the NH_4_Cl prepulse, a prompt intracellular alkalinization occurs due to the rapid passive entry of NH_3_ across the plasma membrane into the cell. Within the aqueous intracellular environment, NH_3_ combines with protons to form NH_4_^+^ and OH^-^ [32, 50], thereby inducing alkalinization. As expected, exposure of PMVECs and PAECs to 20 mM NH_4_^+^ resulted in a rapid increase in pH_i_ by 0.599±0.111 versus 0.500±0.118 pH units, respectively (p = ns; Fig 1A and 1B). Indeed, the extent of alkalinization is thought to be dependent upon the NH_4_Cl concentration and independent of the pH of the external solution; thus, as anticipated, these data reveal the plasma membrane of PMVECs and PAECs are both readily permeable to NH_3_, and each cell type has a similar buffering power to this weak base. During the ammonium prepulse, NH_3_-induced alkalinization continues until intracellular NH_3_ is in equilibrium with extracellular NH_3_; however, the NH_3_-induced alkalinization is offset by a slow gradual acidification due to the influx of NH_4_^+^ through non-specific cation channels and in some cases through NHEs. Once within the cell, NH_4_^+^ dissociates into NH_3_ and H^+^. While the NH_3_ leaves the cell, the H^+^ remains and a slowly progressing, gradual decline in the pH_i_ occurs [23, 32]. In our experiments, there was no difference in the slow decline in pH_i_ between PMVECs and PAEC, in which acidification occurred at a rate of 0.034±0.003 and 0.040±0.002 ΔpH_i_/minute, respectively (red box in Fig 1A and 1B and represented in Fig 1C; p = ns).

**Table 2:** Buffers used during ammonium prepulse experiments. Concentrations are expressed in mM, except pH. *NMNG (N-methyl-D-glucamine).

Reagent, mM	Buffering Solutions			
	HBSS	NH_4_Cl prepulse	CO_2_/HCO_3_^-^ free, Na^+^ free	CO_2_/HCO_3_^-^free, with Na^+^
NaCl	130	110	-	130
NMDG^+^	-	-	118	-
NH_4_Cl	-	20	-	-
KCl	5	5	-	-
MgCl_2_	1.2	1	1	1
CaCl_2_	1.5	1.5	-	-
Glucose	10	10	5	5
HEPES	10	20	10	10
Ca^2+^ gluconate	-	-	1.3	1.3
KH_2_PO_4_	-	-	0.4	0.4
K_2_HPO_4_	-	-	1.6	1.6
Choline Cl^-^	-	-	25	25
pH	7.4	7.4	7.4	7.4

In the second phase of the protocol, the NH_4_Cl prepulse is replaced with a solution containing amiloride, a Na^+^/H^+^ exchange inhibitor (Table 2). The first amiloride-containing solution lacks Na^+^, while in the second amiloride-containing solution, Na^+^ is subsequently added. Following the removal of NH_4_Cl from the external solution, NH_3_ rapidly diffuses out of the cell. Since the driving force for net NH_4_^+^ efflux is less than the previous influx of these ions, the cell loses fewer proton carriers than it has gained. Thus, withdrawal of NH_4_Cl from the medium, results in a decline in pH_i_ to a level lower than before NH_4_Cl exposure. In our experiments, the absolute decrease in pH_i_ following removal of NH_4_Cl was dependent upon the concentration of amiloride in the solution (blue arrow in Fig 1A and 1B and ΔpH_i_ represented in Fig 1D). For example, in PMVECs, exchange of the NH_4_Cl solution to a Na^+^ free-amiloride solution led to a decline in pH_i_ that was greatest at a 400 μM amiloride concentration. The decrease in pH_i_ during the NH_4_Cl-to-amiloride exchange was significantly greater at 400 μM amiloride compared to the decrease in pH_i_ at 40 μM amiloride (Fig 1D). Further, at 400 μM amiloride there was a significantly greater decrease in pH_i_ in PMVECs compared with PAECs. In PAECs, 1 mM amiloride was required to attain the same decrease in pH_i_ as 400 μM amiloride in PMVECs.

During the next phase of the prepulse experiment, in the absence of Na^+^, and in the presence of increasing concentrations of amiloride, there is no recovery from NH_4_Cl-induced-acidification in either PMVECs or PAECs demonstrating the recovery from acidification is dependent upon media sodium (blue box in Fig 1A and 1B, and rates represented in Fig 1E). Once Na^+^ is added back to the external solution, recovery from acidification proceeds in an amiloride dose-dependent manner. In PMVECs, as the dose of amiloride increases, the rate of recovery from acidification in the presence of Na^+^ decreases (green box shown in Fig 1A and rates represented in Fig 1F). At 400 μM amiloride, recovery from acidification in PMVECs is completely inhibited. In PAECs, a similar dose-dependent decrease in recovery from acidification occurs, yet a slow recovery still occurs at 400 μM and is only fully inhibited at 1 mM amiloride (green box shown in Fig 1B and rates represented in Fig 1F). Thus, the amiloride-sensitive recovery from acidification is different between PMVECs and PAECs (40 μM), such that the IC_50_ is left shifted revealing a greater sensitivity to amiloride in PMVECs compared to PAECs (Fig 1F).

Finally, amiloride is removed from the buffer in the presence of Na^+^, and in all cases, the pH_i_ recovers to a pH_i_ similar to baseline (Fig 1A and 1B).

### NHE expression in PMVECs versus PAECs

Recovery from cellular acidification can occur through the actions of various proton and bicarbonate transporters. When bicarbonate is eliminated from the buffer, recovery from acidification is governed by electroneutral alkali cation-H^+^ exchangers, that couple the transfer of H^+^ across the membrane to the counter transport of a cation such as Na^+^ or K^+^. The thirteen Na^+^-H^+^ exchanger orthologues are classified as SLC9A, SLC9B, and SLC9C. These NHEs are differentially expressed at the plasma membrane and organelles along the secretory and endocytic pathways and have been described for their sensitivity to amiloride [25, 51]. In this study, we set out to identify the differential expression of *Slc9a* genes encoding plasma membrane transporters that could account for the difference in recovery from acidification between PMVECs and PAECs; however, in order to validate differential mRNA expression between these two cell types, stable housekeeping or normalization genes as well as the minimum number of reference genes required for reliable normalization was established.

**Fig 2:**
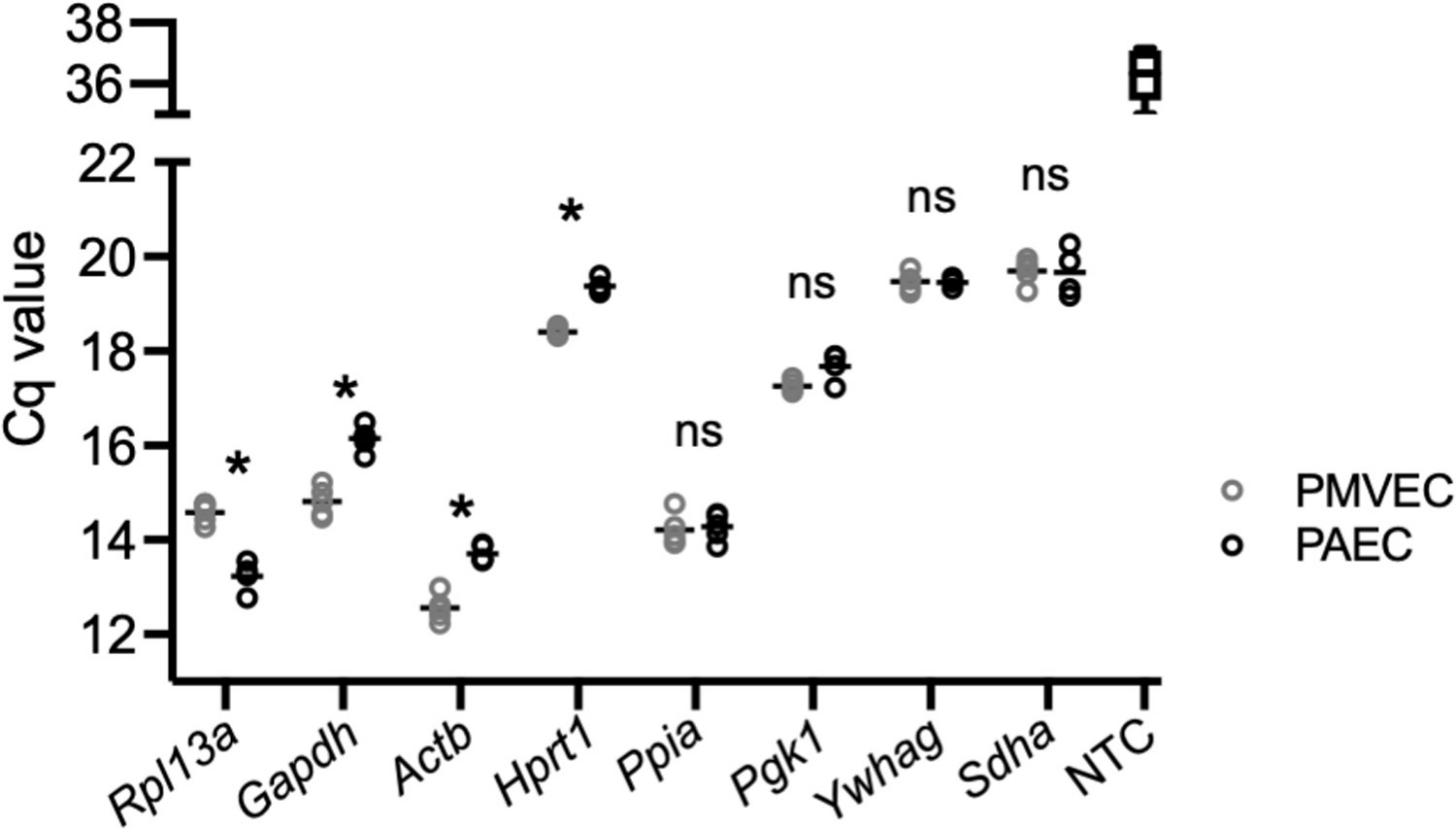
Cq values of housekeeping genes in PMVECs and PAECs. Following qPCR, Cq values for each of the potential normalization genes were compared between PMVECs and PAECs (n = 3–5 different PMVEC and PAEC templates; NTC no template control; *P<0.001 PMVEC versus PAEC; ns not significant).

**Fig 3:**
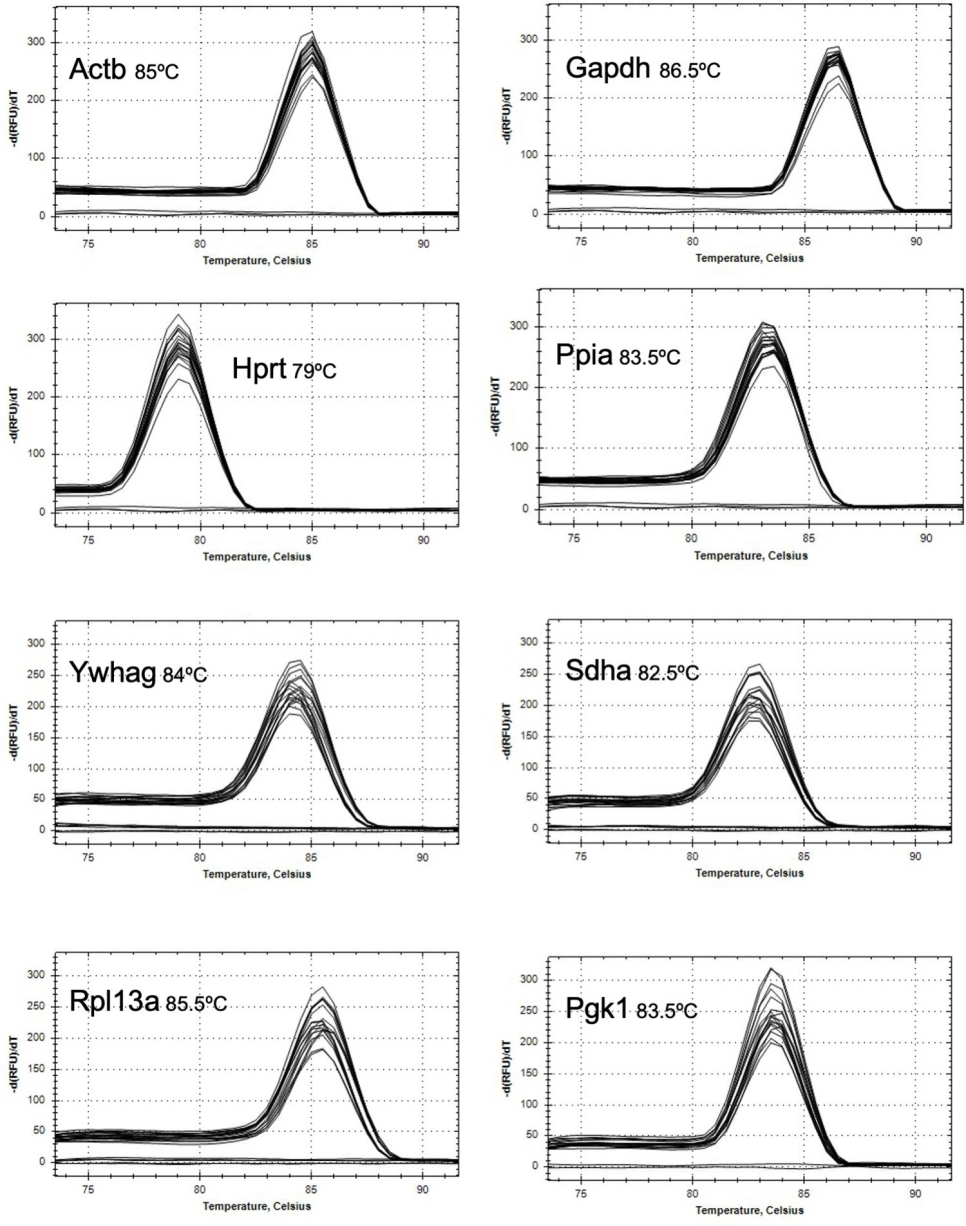
Melt curves demonstrate primer specificity for potential housekeeping genes. Following qPCR reactions, melt curve analysis was performed. Symmetrical single peaks demonstrate unique amplicons and primer specificity. No amplification products were detected in the no-template control reactions.

In order to determine the minimum number of internal control genes for analysis and comparison, eight commonly used reference genes expressed on different chromosomes and belonging to different functional classes were evaluated for stability between PMVECs and PAECs (Table 3). Following RT and qPCR, quantification cycle (Cq) values of the eight reference genes were compared between PMVECs and PAECs (Fig 2). There was a significant difference in the Cq values between PMVEC and PAEC templates with primers for *Actb* (12.56 ± 0.284 in PMVECs versus 13.70 ± 0.171 in PAECs), *Hprt1* (18.404 ± 0.089 in PMVECs versus 19.370 ± 0.137 in PAECs), *Gapdh* (14.81 ± 0313 in PMVECs versus 16.15 ± 0.264 in PAECs) and *Rpl13A* (14.58 ± 0.218 in PMVECs versus 13.23 ± 0.330 in PAECs) suggesting these genes are unsuitable as housekeeping genes between the two cell types. Importantly, there was no significant difference in Cq values between PMVECs and PAECs using primers for *Ppia* (14.206 ± 0.338 versus 14.274 ± 0.286), *Pgk1* (17.254 ± 0.12 versus 17.656 ± 0.311), *Sdha* (19.474 ± 0.196 versus 19.450 ± 0.115), and *Ywhag* (19.694 ± 0.264 versus 19.665 ± 0.512). Sharp and symmetric curves generated during the melting stage of qPCR verified the presence of a single amplification product in each of the reactions with no peaks in the no-template control reactions and demonstrated the specificity of the primers for the eight candidate reference genes in both PMVECs and PAECs (Fig 3). The melting temperature of each amplicon ranged from 79°C (Hprt1) to 86.5°C (Gapdh).

**Table 3:** Candidate reference genes used for RT-qPCR experiments. Gene symbols are represented followed by full gene names, general function of protein and gene accession number.

Gene symbol	Full Gene Name	Function of protein encoded by gene	Accession Number
*Sdha*	Succinate dehydrogenase complex flavoprotein subunit A	Subunit of complex II of the electron transport chain. Transfers electrons from succinate to ubiquinone.	NM_130428.1
*Ywhag*	Tyrosine 3-monooxygenase/tryptophan 5-monooxygenase activation protein, gamma	Adapter protein implicated in regulation of signaling pathways	NM_019376.2
*Pgk1*	Phosphoglycerate kinase 1	Glycolytic enzyme catalyzing conversion of 1,3-diphosphoglycerate to 3-phosphoglycerate	NM_053291.3
*Ppia*	Peptidylprolyl isomerase A	Peptidyl-prolyl cis-trans isomerase involved in protein folding	NM_017101.1
*Hprt1*	Hypoxanthine phosphoribosyltransferase 1	Transferase plays a role in purine nucleotide generation	NM_012583
*Actb*	Beta actin (β-actin)	Cytoskeletal protein	NM_031144.3
*Gapdh*	Glyceraldehyde-3-phosphate- dehydrogenase	Reversible oxidative phosphorylation of glyceraldehyde-3-phosphate during carbohydrate metabolism	NM_017008.4
*Rpl13a*	Ribosomal protein L13A	Ribosomal protein that is a component of the 60s subunit	NM_173340.2

**Fig 4:**
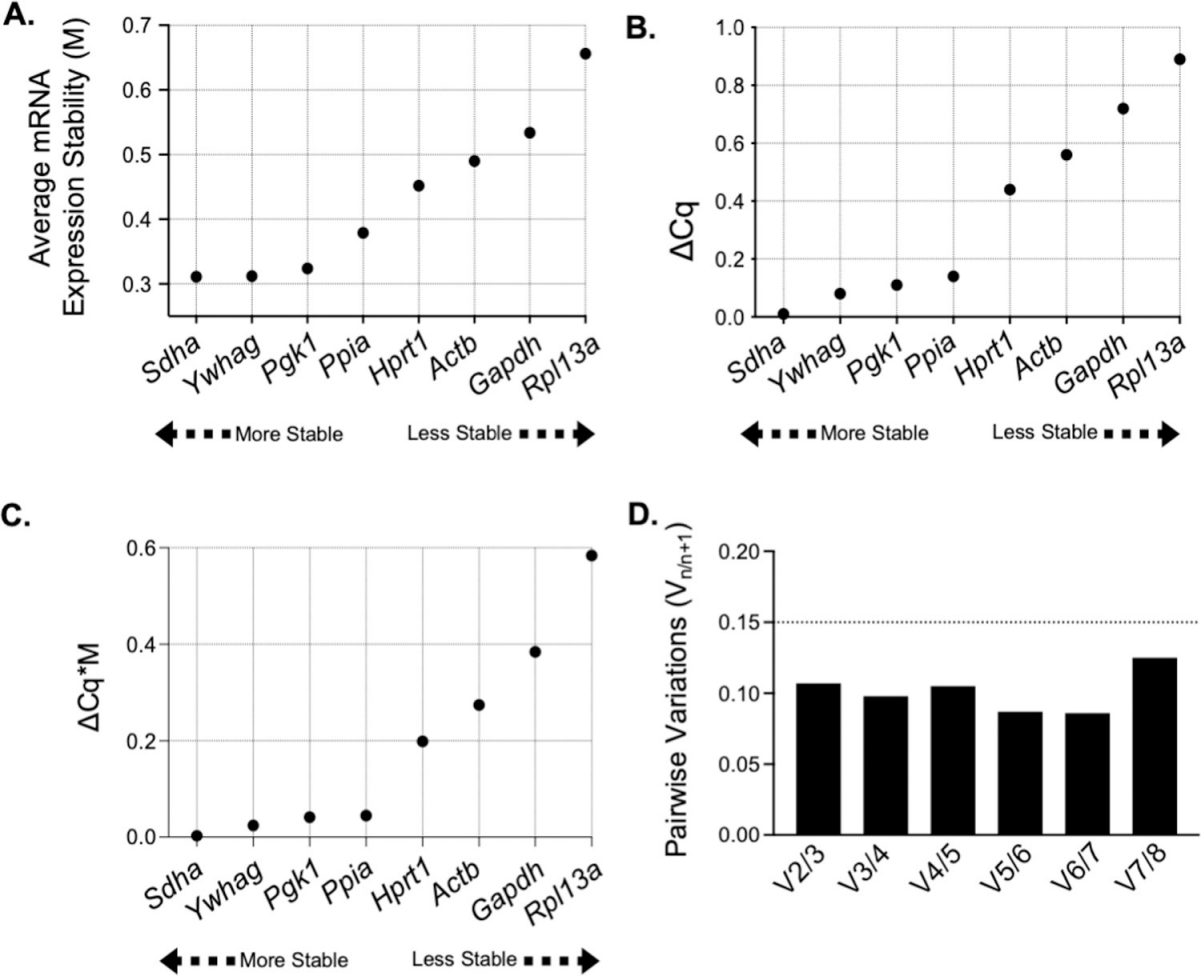
*Sdha* and *Ywhag* selection as normalization genes. (A) *Sdah* and *Ywhag* have the lowest expression stability measure, M, the lowest ΔCq (B) and the lowest ΔCq*M value thereby demonstrating the most stable normalization genes. (D) Using pairwise variation, 2 normalization genes, *Sdah* and *Ywhag*, are sufficient for normalization.

Thus, in order to identify the most stable reference genes, Cq values from qPCR experiments were imported into qBase^PLUS^ for analysis using the geNorm algorithm [44]. This algorithm calculates the expression stability measure (M) for the eight candidate reference genes (Table 3), where lower M values reflect more stable reference genes between samples (i.e. PMVECs and PAECs). *Sdha* and *Ywhag* were the two most stable reference genes with M values of 0.311 and 0.312 respectively (Fig 4A). As additional step to account for possible intergroup variability in mRNA expression between biological samples (i.e. PMVECs and PAECs), geNorm calculated the difference of the average Cq value (ΔCq) of the reference genes between PMVECs and PAECs (Fig 4B). Subsequently, these values are adjusted to calculate a ΔCq*M value [45] (Fig 4C). Smaller ΔCq*M scores represent optimal genes for normalization between sample types. *Sdha* and *Ywhag* remained the two most stable reference genes. Beyond ranking the most stably expressed reference genes, we also defined the number of reference genes required for reliable and accurate normalization of qPCR by calculating the pairwise variation parameter (Vn/n+1) [44]. Since the V-value of V2/3 was 0.107 and below the 0.15 threshold (Fig 4D), only the top two reference genes, *Sdha* and *Ywhag*, were required for reliable normalization and further analysis of the NHE mRNA expression data.

**Fig 5:**
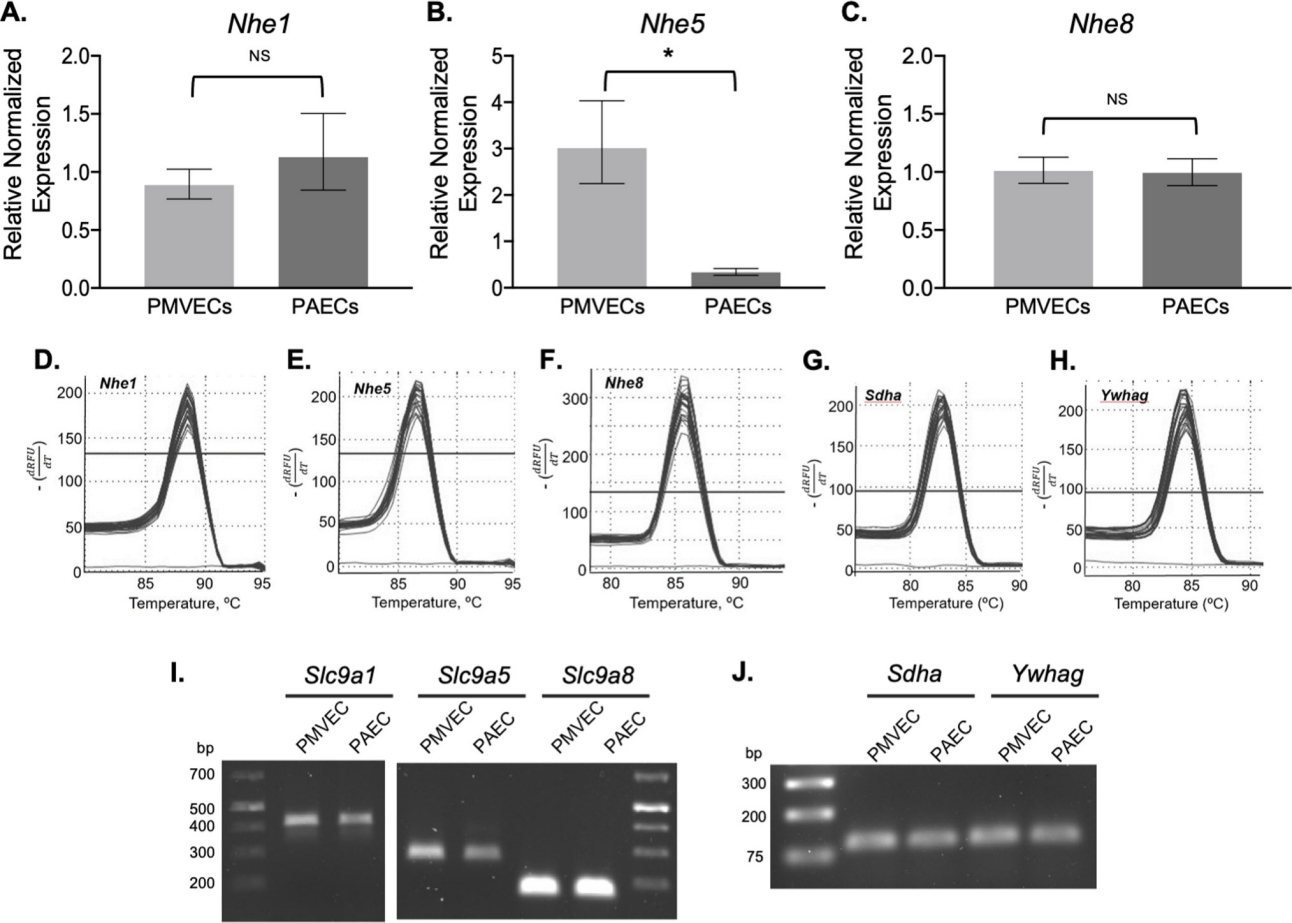
*Nhe5* expression is higher in PMVECs compared with PAECs. q-PCR analysis of PMVECs and PAECs reveals equal expression of NHE1 (A) and NHE8 (C) between PMVECs and PAECs, while NHE5 expression is higher in PMVECs compared to PAECs (B). Values are normalized to *Sdha* and *Ywhag* expression. (D-H) Single symmetrical peaks and single bands on agarose gel (I and J) reveal primer and amplicon specificity. (*P<0.0001 PMVECs versus PAECs; qPCR performed in triplicate, 3–5 separate qPCR experiments were performed each using a unique PMVEC and PAEC template).

**Fig 6:**
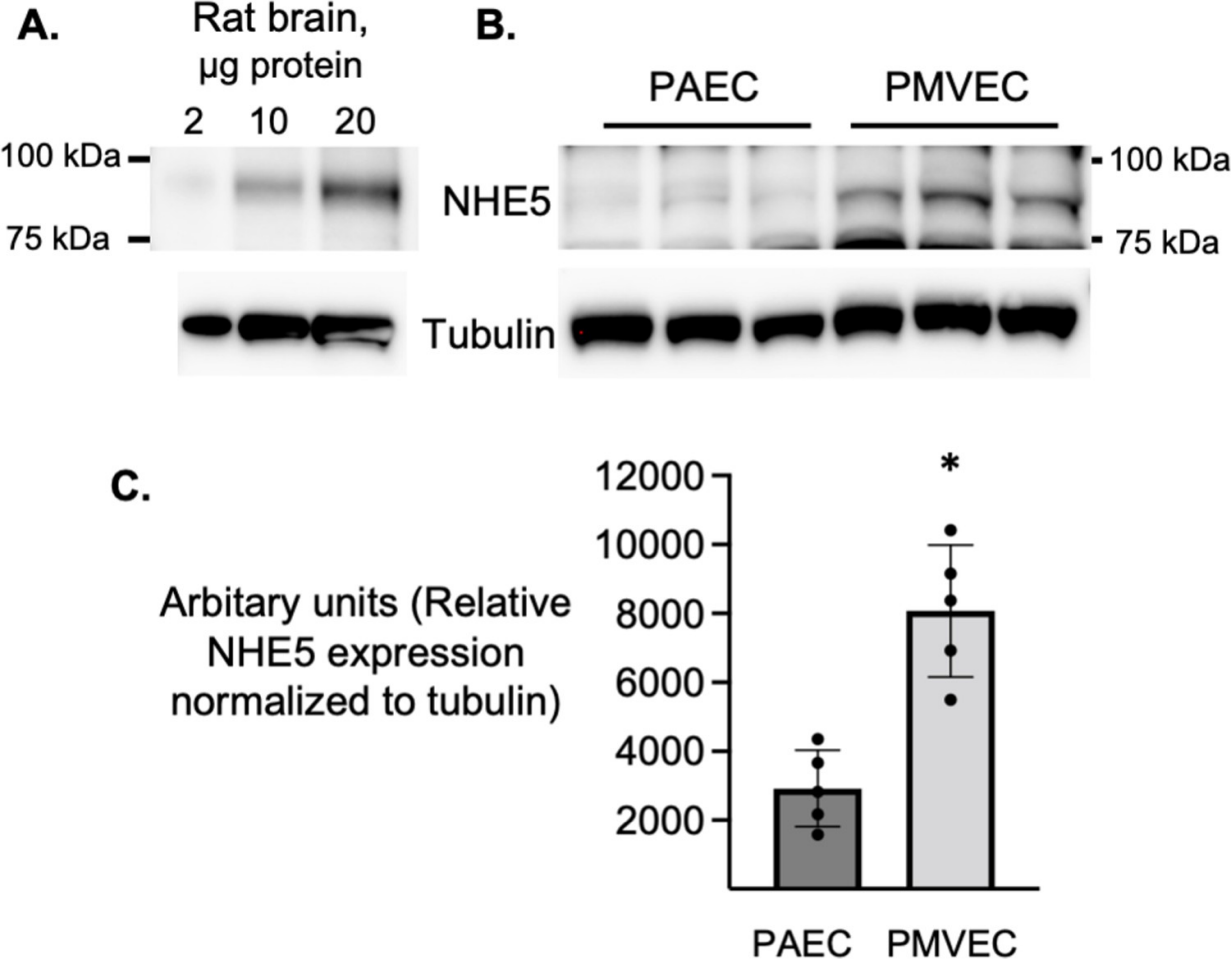
PMVECs cells express NHE5 protein. (A) NHE5 detected in whole brain lysates (B) recognizes a protein band at the same molecular weight in PAECs and PMVECs. Moreover, NHE5 protein expression is greater in PMVECs compared to PAECs. (C) Densitometry summarizing western blots of NHE5 protein expression which is greater in PMVECs versus PAECs. n = 5 *p<0.0001.

Once the carefully selected stable reference genes, *Sdha* and *Ywhag*, had been identified, mRNA expression of membrane-bound NHEs, isoforms 1, 2, 3, 4, 5, and 8, was determined using qRT-PCR and *Rattus norvegicus* specific primers (Table 4). A threshold Cq cutoff value of 30 cycles was used to determine specific and significant mRNA expression. Using *Sdha* and *Ywhag* as the two reference genes for normalization, RT-qPCR revealed similar expression of *Slc9a1* in PMVECs and PAECs, p = 0.17 (Fig 5A). Average Cq values were 25.40 ± 0.18 and 25.14 ± 0.24 respectively. *Slc9a5* expression was greater in PMVECs vs PAECs, p<0.001 (Fig 5B). Average Cq values were 28.85 ± 0.31 and 32.16 ± 0.43 respectively. *Slc9a8* was expressed similarly in PMVECs and PAECs, p = 0.777 (Fig 5C) with average Cq values of 24.33 ± 0.12 and 24.41 ± 0.26 respectively. In both PMVECs and PAECs, primers targeting *Slc9a2*, *3*, and *4* gave Cq values above 30 cycles, yet in stomach and kidney which acted positive controls, Cq values were in the 15–18 range demonstrating primer efficiency. Primer products for *Slc9a1*, *5*, and *8*, positive controls for *Slc9a2*, *3*, and *4* and reference genes *Sdha*, and *Ywhag* had a single symmetric peak on dissociation curve analysis (Fig 5D–5H). To further validate primer specificity, agarose gel electrophoresis of qPCR products revealed a single amplicon at the expected size (Fig 5I and 5J). Thus, using a dual normalization gene approach, our data reveal both PMVECs and PAECs express equal levels of *slc9a1* and *slc9a8* mRNA, while PMVECs express higher levels of *slc9a5* mRNA compared to PAECs. Finally, using an antibody against NHE5 that recognizes a band of the same molecular weight in rat brain lysates (Fig 6A), western blot analysis corroborated the mRNA findings and confirmed NHE5 protein levels are higher in PMVECs versus PAECs (Fig 6B and 6C).

**Table 4:** Primer sequences, amplicon length and accession number for *slc9a1-5*, *8* genes expressing plasma membrane NHEs.

Gene	Protein name	Primer sequence (5’→3’)	Amplicon length (bp)	Accession Number
*Slc9a1*	Sodium-hydrogen exchanger 1 (NHE1)	F: TCTGCCGTCTCAACTGTCTCTA	422	NM_012652.1
		R: CCCTTCAACTCCTCATTCACCA		
*Slc9a2*	Sodium-hydrogen exchanger 2 (NHE2)	F: GCAGATGGTAATAGCAGCGA	312	NM_001113335.1
		R: CCTTGGTGGGGGCTTGGGTG		
*Slc9a3*	Sodium-hydrogen exchanger 3 (NHE3)	F: GGAACAGAGGCGGAGGAGCAT	322	NM_012654.1
		R: GAAGTTGTGTGCCAGATTCTC		
*Slc9a4*	Sodium-hydrogen exchanger 4 (NHE4)	F: TTCCCCCGAAGACGTGGAATCC	255	NM_173098.1
		R: GCTGGCTGAGGATTGCTGTAA		
*Slc9a5*	Sodium-hydrogen exchanger 5 (NHE5)	F: ATTGACACAGTACGCAGTGG	274	NM_138858.1
		R: TAGCCACACCATCCTTCTTC		
*Slc9a8*	Sodium-hydrogen exchanger 8 (NHE8)	F: AAGCCTATTCTTCCGGTGCAGACA	169	NM_001025281.1
		R: AGAGAAACAACAGCCACGCTCTCA		

## Discussion

This study contributes to the growing understanding that functional heterogeneity exists between endothelial cells from different vascular segments of the lung. For example, PMVECs exhibit unique recovery from cellular acidification in the presence of amiloride compared PAECs. Absence of HCO_3_^-^ from the buffering system, permits the resolution of NHE activity during recovery from an acid load. Specifically, these findings reveal amiloride induces a greater decline in pH_i_ in PMVECs compared to PAECs following removal of the ammonium prepulse. Further, these studies reveal PAECs require higher concentrations of amiloride to inhibit recovery from acidification compared to PMVECs. The reason for this unique amiloride sensitivity between PAECs and PMVECs suggests higher NHE activity. Alternatively, since the sensitivity of NHE to amiloride is isoform specific, ranging from IC_50_ 1.6 μM for NHE1 [52] to 813 μM for NHE4 [53], this could reflect differential NHE isoform expression in PAECs compared to PMVECs. Importantly, using housekeeping/normalization genes that remain constant between endothelial cells from the different vascular segments, these studies reveal similar mRNA expression of NHE1 and NHE8 between PMVECs and PAECs, but a significant 3-fold greater expression of NHE5 in PMVECs versus PAECs, and are the first to describe NHE5 and NHE8 in the pulmonary endothelium. At the protein level, NHE5 expression is also greater in PMVECs compared to PAECs.

Techniques used to detect NHE activity have changed over time. An amiloride-sensitive NHE transport system was first documented in endothelium using a sodium depletion technique to examine sodium fluctuations in microvessels isolated from the blood brain barrier of rats [54] and endothelial cells isolated from bovine aorta [55]. In subsequent studies, rather than detecting fluctuations in radiolabeled sodium, transitions in pH_i_ were monitored in BCECF-loaded cultured bovine aortic endothelial cells [56]. These studies revealed NHE was the major mechanism of pH_i_ recovery from propionate-induced cellular acidification, which was inhibitied by 10 μM 5-(n,n-hexamethylene)amiloride. Within lung endothelium, Cutaia and Parks induced intracellular acidification using a sodium-free nigericin technique followed by albumin scavenge [28–30]. BCECF was used to monitor pH_i_ recovery from acidification in the absence of bicarbonate in both bovine pulmonary artery and microvascular endothelial cells. Throughout this series of studies, recovery from acidification was dependent upon sodium and inhibited by the amiloride analogue methylisobutylamiloride (10 μM). Further, oxidant stress alone or in combination with hyperoxia attenuated the rate of recovery from acidification in PAECs, while hypoxia similarly decreased the rate of recovery from acidification in both PAECs and PMVECs. The studies presented here are the first to employ the now commonly used and preferred ammonium prepulse technique for cellular acidification. Utilizing this technique in combination with BCECF measurements of pH_i_, we demonstrate the unique amiloride-dose dependent recovery from acidification between pulmonary endothelial cells derived from different vascular segments. Future studies will examine the amiloride-sensitivity of pulmonary vein endothelial cells and explore whether various stimuli perturb the dynamics of this process.

NHE1 is constitutively expressed in many cell types and tissues. Indeed, NHE1 mRNA has been documented in pulmonary endothelial cells, and in accordance with our finding, this group were unable to detect NHE2, -3, or -4 [30]. In contrast, NHE1, -2, -3, and -4 have been detected in rat brain capillary endothelial cells while other groups detected NHE5 in rat brain endothelium and whole lung tissue [57–60]. Consistent with many transporters, very low levels of NHE2-5 were reported [58]. Amiloride binds to three residues in the putative transmembrane domain of certain NHE isoforms, such as NHE1 [51], giving rise to specific isoform sensitivity [24]. While NHE1 and 2 have similar sensitivity to amiloride (rat NHE1 IC_50_ 1.6 μM [52] and 5.3 μM in another study [53]), NHE3 has an IC_50_ 100–300 μM [52, 53, 61] and NHE4 IC_50_ 813 μM [53]; thus, NHE3 and -4 are 60 and 160 times less sensitive to amiloride than NHE1, respectively. An IC_50_ of 20μM has been reported for human NHE5 [62]. In our studies, PMVECs have greater sensitivity to amiloride than PAECs. To explain this difference in amiloride sensitivity between the two cell types, we expected to detect expression of the less amiloride-sensitive NHE isoforms, NHE3 or -4, in PAECs; however, we were unable to detect the expression of mRNA for either NHE3 or -4 in either cell type. Further, since the IC_50_ for amiloride of NHE5 is 20 μM, the lack of NHE5 expression in PMVECs, does not resolve the amiloride insensitivity compared to PAECs.

This is the first report of NHE5 in pulmonary endothelial cells. NHE5 is enriched in rat and human brain [60, 63, 64] where it is implicated in learning and memory [65]. Further, NHE5 is also detected in sperm [66]. This transporter is associated with both the plasma membrane and recycling endosomes [67] and colocalizes with RACK1 and beta integrin to regulate cell-matrix adhesions [68]. NHE5 has been implicated in tumor growth, cell proliferation and invasion through regulation of growth factor signaling [69]. While NHE5 has been detected in brain endothelial cells, its function is not resolved [58, 59]. In line with the recently described roles for NHE5, future studies will examine whether NHE5 expression supports the pro-proliferative and angiogenic phenotype of PMVECs compared to PAECs. Further, since PMVECs utilize aerobic glycolysis to produce ATP and acidify the media, whereas PAECs predominantly rely oxidative phosphorylation with little change to media pH during proliferation to form a confluent monolayer [15], NHE5 may be important in maintaining cellular pH in PMVECs.

NHE8 is expressed at both the plasma membrane and intracellular compartments. Expression of this NHE isoform has recently been described in lung tissue and the apical domain of alveolar epithelial cells isolated from rat. Interestingly, NHE8 expression in A549 cells is decreased following Ang II treatment and is altered in a rat model of congestive heart failure [70]. Here, we report NHE8 in both PMVECs and PAECs, which is the first finding of this isoform in the endothelium.

Collectively, these studies reveal a unique recovery from acidosis between pulmonary capillary endothelial cells (i.e. PMVECs) and pulmonary endothelial cells lining conduit vessels (i.e. PAECs). Here we confirm the expression of NHE1 in both PMVECs and PAECs, and reveal the expression of the novel isoform, NHE8, also in both cell types. Using suitable housekeeping genes for comparison of mRNA at a quantitative level between the two different cell types, NHE5 expression is higher in PMVECs compared to PAECs. Currently, while NHE5 and NHE8 have been reported in lung tissue [60, 70], the physiological significance of NHE5 and NHE8 expression in pulmonary endothelial cells is not resolved and the study of future investigations.

## Supporting information

S1 Raw images (PDF)

S2 Raw image (TIFF)
