# Supplementary figures and images for: Pulmonary endothelial cells from different vascular segments exhibit unique recovery from acidification and Na+/H+ exchanger isoform expression
Source: PLoS One. 2022 May 3;17(5):e0266890. doi: 10.1371/journal.pone.0266890 (PMC9064095; doi:10.1371/journal.pone.0266890)

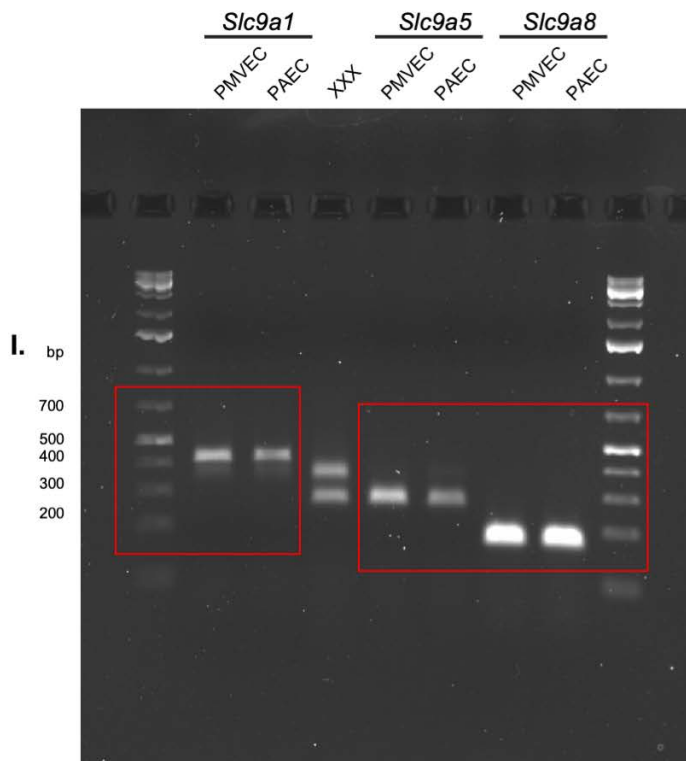

Gels shown in Fig 5I

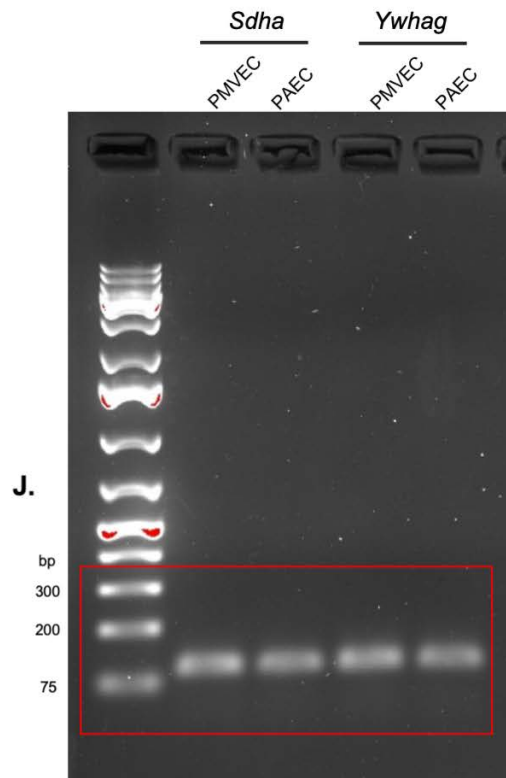

Gels shown in Fig 5J

Supplemental Figure 1.

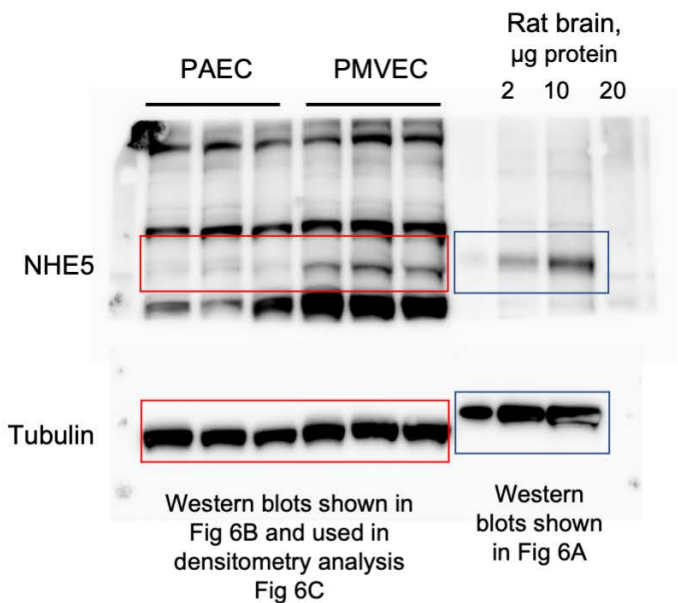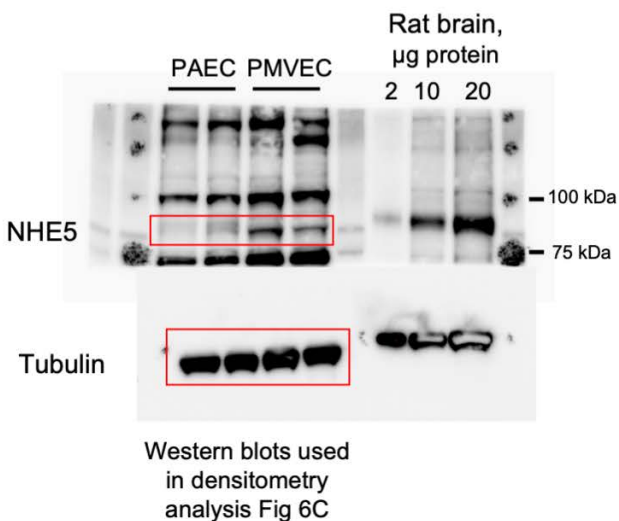

Supplemental Figure 2.

Supplement: S1 Raw images — (PDF) [file pone.0266890.s001.pdf]

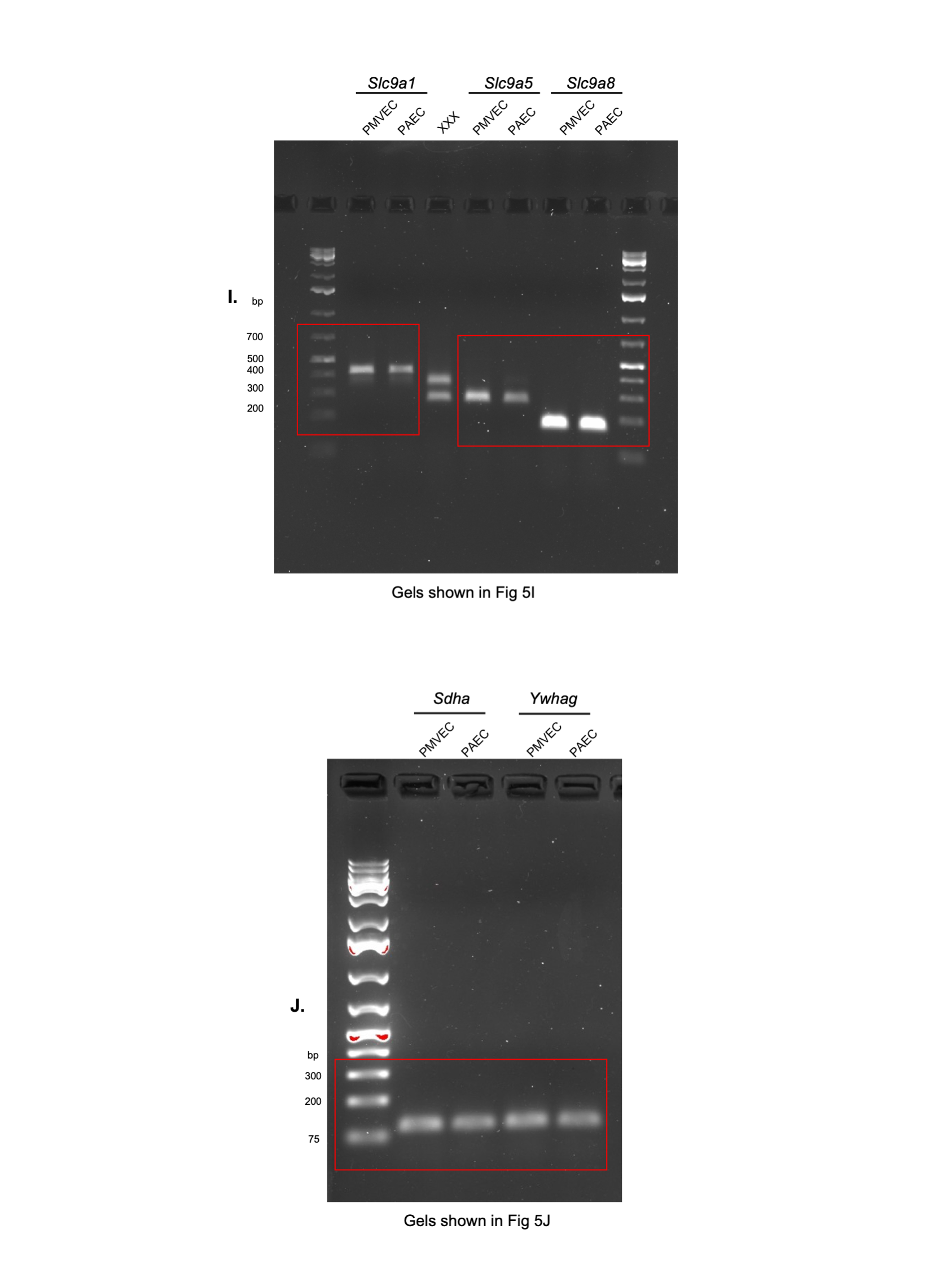

Supplement: S2 Raw image — (TIFF) [file pone.0266890.s002.tiff]
